# From Conflict to Care - Telemedicine Utilization During Wartime: A Retrospective Cohort Study

**DOI:** 10.1007/s10916-025-02220-0

**Published:** 2025-06-17

**Authors:** Sarah Sberro-Cohen, Moriah E. Ellen

**Affiliations:** 1 Department of Health Policy and Management, Faculty of Health Sciences, School of Public Health, Ben-Gurion University of the Negev, Beer- Sheva, Israel; 2 Maccabi Healthcare Services, Southern District, Omer, Israel; 3 Institute of Health Policy, Management and Evaluation, University of Toronto, Ontario, Canada

**Keywords:** Telemedicine, Healthcare Utilization, War, Digital Health, Remote Consultation, Crisis Healthcare

## Abstract

**Background:**

Armed conflict poses severe challenges to healthcare delivery, requiring rapid adaptation. This study evaluates how telemedicine enabled continuity of care during the October 7, 2023, war in Israel, and assess regional and service-specific utilization patterns in relation to conflict intensity.

**Methods:**

A retrospective cohort study of 7.19 million healthcare interactions from an Israeli HMO covering one-third of Israel’s population. The study compared three periods: (T0) the first month of the war, (T1) the month before, and (T2) the same period last year. Interactions included visits and inquiries in primary care, secondary care, mental health, and allied health services. Data were categorized by service type and geographic conflict zones. Chi-square tests and effect sizes assessed trends.

**Results:**

Telemedicine utilization increased significantly during the war, especially in primary conflict zones (13–20%, *p* < 0.01). Remote consultations in mental health tripled (10–30%, *p* < 0.01), and nutrition services reached the highest telemedicine adoption (27–52%, *p* < 0.01). Family medicine, pediatrics, and gynecology also showed significant increases. Digital inquiries surged in family medicine but declined in pediatrics.

**Conclusion:**

This study offers timely insights into telemedicine’s role in maintaining access during armed conflict within a digitally advanced system. By examining service utilization across medical domains and conflict zones, it highlights how remote care supports system adaptability in crises. Notably, patient satisfaction remained high, suggesting telemedicine preserved access and perceived care quality. Findings may inform digital health planning to strengthen continuity, equity, and resilience in future emergencies.

## Introduction

The Israeli healthcare system has demonstrated resilience in the face of numerous challenges, particularly during times of conflict [1–3]. The war that began on October 7, 2023, created an unprecedented emergency that required rapid system-wide adaptation. Unlike previous conflicts, this war was characterized by its intensity, immediacy, and widespread civilian impact, leading to mass displacement and psychological trauma [4]. Armed conflict threatens public health, disrupts healthcare infrastructure, and reduces the availability of healthcare workers—factors that contribute to critical workforce shortages [5]. During wartime, heightened danger reduces healthcare personnel’s willingness to report to work, increasing absenteeism by 73% [2]. In such crises, rapid reorganization is essential to address both physical injuries and escalating mental health needs [6, 7]. Maintaining accessible, effective, and affordable healthcare is critical, even under disrupted conditions [8]. Conflict undermines care continuity by damaging infrastructure and limiting access to services [8, 9]. In health policy, “access” refers to the extent to which services meet patient needs, based on five key dimensions: availability, accessibility, accommodation, affordability, and acceptability [9]. Addressing gaps in access and maintaining service delivery infrastructure is essential for safe, equitable care. When in-person access is unsafe or unavailable, alternative modes of delivery, such as remote service models, are necessary [10].

Telemedicine, as defined by the World Health Organization (WHO), refers to the use of digital technologies to provide care at a distance, supporting diagnosis, treatment, prevention, and patient monitoring [11, 12]. Its adoption during emergencies is well documented, with evidence of high satisfaction among both patients and providers despite technical limitations [13]. Israel’s national health insurance law mandates enrollment in one of four competing health maintenance organizations (HMOs) [14]. Israel has long been a leader in digital health among OECD countries, offering broad access to telemedicine services, especially during national emergencies such as the COVID-19 pandemic [15]. Globally, telemedicine is increasingly recognized as an essential component of routine care as well as crisis response [16, 17]. Though some still prefer face-to-face care [18, 19], telemedicine provides a lifeline in hazardous situations, such as war zones or natural disasters [20].

This study assesses the impact of the October 2023 war on healthcare utilization within Israel’s HMO system. It focuses on the shift from in-person to remote care, examining trends in primary, secondary, and allied health services across conflict zones, while evaluating changes in digital and administrative inquiries as alternative access points.

## Methods

A retrospective cohort study assessed the war’s impact on healthcare utilization in Israel’s community healthcare. The study population included all recorded visits and inquiries from Maccabi Healthcare Services, one of Israel’s four HMOs. Given that Maccabi covers ≈ 2.75 million insured members (27.5% of Israel’s insured population), operates ≈ 400 community medical centers across all districts, and its age-band distribution closely mirrors national figures (15–35 y: 27.2% vs. 27.2%; ≥ 65 y: 12.6% vs. 13%) [21], the study cohort can be considered broadly representative of the Israeli population with respect to demographic structure.

### Study Design and Data Collection

The analysis covered three periods: T0 (war period): The first month of the war, October 7– November 7, 2023. T1 (pre-war): The month before the war, September 7– October 6, 2023 coinciding with Jewish holidays, during which two weekday holidays reduced healthcare availability. T2 (corresponding month period in the previous year): October 7– November 7, 2022 (no holidays). Each comparator period spans the same measurement window, ensuring equal duration across T0, T1, and T2. The study focused specifically on the first month of the war (T0) because this period represents the initial shock phase, marked by widespread national trauma, public fear, and profound disruption to daily life and service access. During this time, many areas experienced direct attacks, and the presence of terrorists inside Israeli territory created an acute sense of insecurity. This timeframe captures the healthcare system’s immediate adaptive response, before stable emergency routines were established or the population adjusted to the evolving conditions. As such, it offers a unique opportunity to examine system resilience and real-time shifts in healthcare delivery under extreme uncertainty.

Districts were classified into three conflict zones according to geographical location and conflict intensity: (1) Primary Conflict Zone: The Southern District, the epicenter of the conflict, experienced frequent attacks, and severe disruptions due to proximity to Gaza. (2) Secondary Conflict Zone: The Northern District, under heightened alert due to threats along Israel’s northern borders. (3) low conflict zones: Central, Jerusalem, Sharon, and Shephelah, which experienced minimal direct conflict but were affected by the national state of emergency.

The dataset included 7.19 million recorded interactions across various healthcare services, including primary care (family medicine, pediatrics, gynecology), secondary care (orthopedics, ENT- Ear, Nose, and Throat, dermatology, ophthalmology), and essential services (nursing, physiotherapy, nutrition, mental health). Healthcare utilization was categorized by mode of delivery, including digital inquiries via the HMO’s mobile app or website for consultations, prescriptions, and medical certificates; remote visits conducted through video or phone consultations; administrative digital inquiries related to service requests, appointment scheduling, and document retrieval; and in-person visits.

### Statistical Analysis

Descriptive statistical analyses were performed to compare healthcare utilization across the three time periods, stratified by service type, conflict zone, and mode of delivery. Differences in service utilization were assessed using Chi-square tests, while effect sizes were calculated to evaluate trends in telemedicine adoption. A p-value of less than 0.05 was considered statistically significant. Statistical analyses were conducted using IBM SPSS Statistics (version 29.0).

## Results

### Overall Telemedicine Utilization

The study analyzed 7.19 million visits and inquiries, revealing significant shifts in healthcare service utilization during the war compared to routine times. Telemedicine use increased across all conflict zones, with the most pronounced rise in primary conflict zones, where remote visits peaked. This trend coincided with a decline in in-person visits, highlighting the shift toward remote care. Table 1 presents the ratio of remote to in-person service utilization across conflict zones. During the war period, all districts combined saw an increase in remote service utilization from 10% in the previous year to 13%. The primary conflict zones, which included areas under heavy bombardment and significant civilian displacement, showed the highest shift towards remote services, with the ratio rising to 20% during the war.

**Table 1:** Remote vs. In-person visits by conflict zones– all services

Conflict zone	T0 - War period (*N*)	T1 - Pre-war period (*N*)	T2 - Corresponding period in the previous year (*N*)
All HMO’s districts	13% (229286)	11% (188510)	10% (182983)
Primary conflict zones	20% (49440)	13% (36174)	13% (35875)
Secondary conflict zones	11% (39521)	9% (32250)	9% (31280)
low conflict zones	12% (140325)	10% (120086)	10% (115828)

### Telemedicine Visits Utilization in Primary and Secondary Medicine

In primary conflict zones, telemedicine use in primary and secondary medicine increased significantly during the war (Table 2). Family medicine led this trend, with telemedicine visits rising from approximately 16% in both the pre-war period (T1) and the previous year (T2) to over 25% during the war (T0) (*p* < 0.01). Pediatrics also showed a notable increase, from nearly 17% to over 23% (*p* < 0.01). Gynecology visits nearly doubled, rising from just over 7% (T1) to nearly 14% (T0) (*p* < 0.01). Secondary medicine saw a smaller but significant increase in the primary conflict zones, from around 3.44% (T2) to 7.50% (T0) more than doubling the previous percentages (*p* < 0.01). In secondary and low conflict zones, increases were more modest. Family medicine and pediatrics maintained the highest telemedicine utilization rates, though the growth was less pronounced than in primary conflict zones.

**Table 2:** Telemedicine utilization in primary and secondary medicine (percentage of total visits)

Healthcare service	Conflict zone	War period T0	Pre-war period T1	Last year corresponding period T2
Family medicine	Primary conflict zones	25.34%	16.54%	16.61%
	Secondary conflict zones	13.58%	12.40%	12.28%
	low conflict zones	15.78%	14.43%	13.49%
Pediatrics	Primary conflict zones	23.31%	16.86%	16.76%
	Secondary conflict zones	10.51%	10.20%	10.61%
	low conflict zones	14.35%	12.57%	13.63%
Gynecology	Primary conflict zones	13.74%	7.01%	6.29%
	Secondary conflict zones	7.13%	6.58%	5.92%
	low conflict zones	7.87%	5.97%	4.96%
Secondary medicine	Primary conflict zones	7.50%	3.88%	3.44%
	Secondary conflict zones	2.86%	2.40%	2.44%
	low conflict zones	4.98%	4.32%	4.10%

### Telemedicine Utilization in Nursing and Health Professionals

Nursing services experienced a notable increase in telemedicine usage (Table 3), reached over 16% during the war, up from approximately 14% in the pre-war period and nearly 11% in the previous year (*p* < 0.01), indicating a shift towards remote nursing care as access to healthcare facilities became more difficult. Physiotherapy also increased, though more modestly, from just over 1% in the pre-war period to nearly 3% during the war (*p* < 0.01), indicating adaptation despite its hands-on nature.

Nutrition services showed the most dramatic increases, with remote visits more than doubling from reaching over 52% during the war, up from approximately 27% in the pre-war period and nearly 24% in the previous year (*p* < 0.01). This marks the only service where remote visits surpassed in-person visits, highlighting a major shift in care delivery. In secondary and low conflict zones, increases were more modest, with nursing and allied health services maintaining the highest telemedicine utilization rates.

**Table 3:** Telemedicine utilization in nursing and health professional services (percentage of total visits)

Healthcare service	Conflict zone	War period T0	Pre-war period T1	Last year corresponding period T2
Nursing	Primary conflict zones	16.65%	13.92%	10.99%
	Secondary conflict zones	12.85%	9.80%	7.30%
	low conflict zones	9.19%	10.03%	6.96%
Physiotherapy	Primary conflict zones	2.96%	1.29%	1.12%
	Secondary conflict zones	0.22%	0.34%	0.17%
	low conflict zones	0.41%	0.39%	0.41%
Nutrition	Primary conflict zones	52.64%	27.36%	23.94%
	Secondary conflict zones	30.57%	19.84%	18.52%
	low conflict zones	31.45%	15.88%	14.82%

### Telemedicine Utilization in Mental Health Services

In primary conflict zones, mental health services exhibited a substantial increase in telemedicine utilization (Table 4), during the war period (T0). The proportion of remote consultations for mental health services more than doubled, rising by over 20% compared to the pre-war period (T1). Specifically, telemedicine visits accounted for approximately 30.39% of all mental health consultations during the war period, up from about 10.14% in the pre-war period and around 11.11% in the corresponding period from the previous year (T2). The dramatic increase in telemedicine utilization for mental health services during T0 compared to T1 and T2 (*p* < 0.01).

In secondary and low conflict zones, the changes were more modest. In secondary conflict zones, telemedicine visits increased slightly from 6.26% in T1 to 7.80% in T0, while in low conflict zones, telemedicine increased from 9.53% in T1 to 19.12% in T0 (*p* < 0.01). While statistically significant, these increases were less pronounced than in primary conflict zones.

**Table 4:** Telemedicine utilization in mental health services (percentage of total visits)

Healthcare service	Conflict zone	War period T0	Pre-war period T1	Last year corresponding period T2
Mental health	Primary conflict zones	30.39%	10.14%	11.11%
	Secondary conflict zones	7.80%	6.26%	7.64%
	low conflict zones	19.12%	9.53%	9.09%

### Trends in Digital Inquiries

**Fig. 1:**
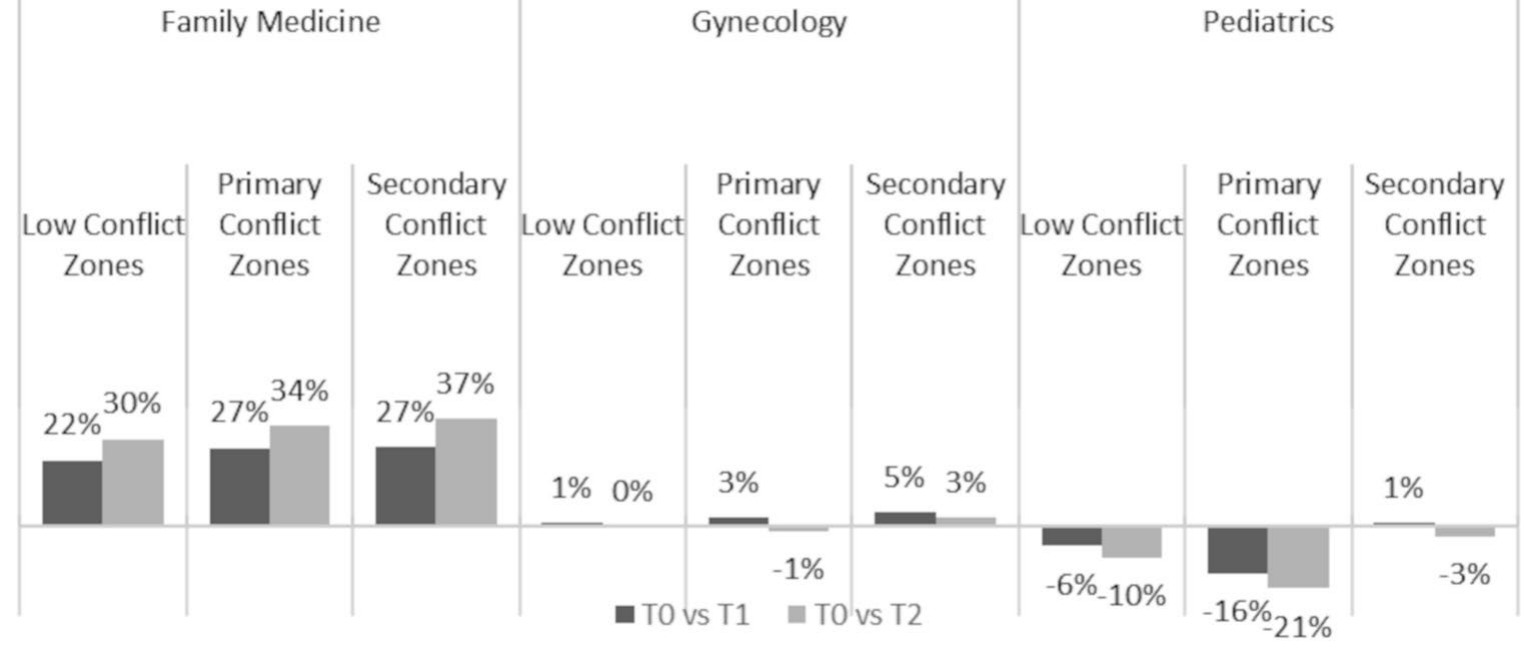
Percentage change in telemedicine utilization during war vs. routine

Digital inquiries, defined as asynchronous consultations via the HMO’s mobile app or website for physician advice, prescription requests, and medical certificates, showed notable changes during the war period (T0) compared to routine times (T1, T2) (Fig. 1). The most substantial increases were in the primary and secondary conflict zones, particularly within family medicine services. Family medicine in primary conflict zones showed a remarkable increase, with digital inquiries rising by 26.58% compared to T1 and 34.04% compared to T2. Secondary conflict zones experienced increases of 26.91% compared to T1 and 36.52% compared to T2 in family medicine digital inquiries. Gynecology services exhibited more modest changes, with slight increases in both primary and secondary conflict zones. For example, in primary conflict zones, digital inquiries increased by 3.23% from T1 to T0, though there was a slight decrease of 1.40% when compared to T2. In contrast, pediatrics displayed a decrease in digital inquiries in low and primary conflict zones during the war period, with reductions of up to 20.67% compared to T2.

### Administrative Inquiries Utilization

During the war period (T0), there was a noticeable increase in administrative inquiries across most healthcare services compared to both the pre-war period (T1) and the corresponding period from the previous year (T2). Family medicine saw the most significant rise, particularly in primary conflict zones, where inquiries more than doubled from T1 and T2. This sharp increase reflects the heightened administrative demands faced by family medicine during the conflict. Gynecology also experienced a moderate increase in administrative inquiries, with the primary conflict zones again showing the most substantial rise. Mental health services saw one of the most dramatic increases, especially in primary conflict zones where inquiries tripled compared to T1 and were more than five times higher than T2. This surge underscores the critical administrative support needed in mental health services during the war. Pediatrics followed a similar pattern, with significant increases in administrative inquiries, particularly in primary conflict zones. Here, inquiries rose by over 74% compared to T1 and more than doubled compared to T2. In contrast, secondary care showed a slight decrease in administrative inquiries during the war period compared to T1, with only a minor increase over T2.

## Discussion

This study offers valuable insights into the impact of the October 7, 2023, war on healthcare service utilization within Israel’s HMO system. The findings indicate a marked shift from in-person visits to telemedicine, particularly in regions most severely affected by the conflict. This shift reflects a broader global trend observed in conflict-affected areas, where healthcare systems need to adapt rapidly to ensure continuity of care under challenging conditions [22]. While the phenomenon of increased telemedicine use during crises is well documented—especially during the COVID-19 pandemic—the context of sudden armed conflict presents a different type of disruption. Unlike a prolonged health emergency, war involves immediate physical danger, population displacement, and damage to healthcare infrastructure. These unique conditions make the Israeli experience during October 2023 a distinctive case that expands the global understanding of digital health adaptation in extreme settings. Although the current analysis does not include data on diagnoses or clinical outcomes, it contributes a system-level view of how various services—such as mental health, nutrition, family medicine, and gynecology—adapted to the use of telemedicine across conflict zones. These findings highlight which services were more flexible or in higher demand during the crisis, offering early signals for where digital readiness and emergency planning should be prioritized.

One of the noteworthy findings of this study is the increase in telemedicine utilization across all healthcare services during the war. This trend was most prominent in primary conflict zones, where telemedicine became a crucial mode of consultation. The services that showed the most significant increases in telemedicine use included nutrition, mental health services, family medicine, and pediatrics.

Among these, nutrition services saw the most pronounced increase in telemedicine utilization. The proportion of telemedicine visits for nutrition more than doubled during the war period, a statistically significant change that underscores the shift toward remote nutritional consultations. This finding aligns with existing evidence that telehealth, including tele-nutrition, plays a crucial role in delivering health services remotely, particularly when distance and accessibility are critical factors. Tele-nutrition has been effective in promoting healthy lifestyles, self-monitoring, and behavioral change, which is especially valuable during crises [23, 24].

The war also revealed a significant demand for mental health services, with telemedicine playing a vital role in meeting this need. The rise in mental health telemedicine reflects the psychological toll of the conflict and the essential role of remote care. Similar patterns were seen during COVID-19 pandemic. Telemedicine usage for mental health has proven effective to manage conditions like PTSD (Post-Traumatic Stress Disorder), anxiety, and depression, which are prevalent during such times [25].

As expected, there was a corresponding decline in in-person visits during the war, especially in the primary conflict zones. This shift underscores the adaptability of healthcare systems in crisis situations, where digital platforms are leveraged to provide continued care under challenging circumstances [26]. Highlighting the crucial role that telemedicine plays in maintaining healthcare access when traditional in-person services are disrupted [27]. Moreover, the study noted an increase in digital inquiries and administrative support during the war, particularly in the primary conflict zones. The rise in administrative demands suggests that the healthcare system responded effectively to the evolving needs of the population under stress, ensuring that essential services remained accessible through digital means.

While this study did not include outcome-based quality measures or patient satisfaction scores as primary endpoints, supplemental monthly patient experience survey data conducted by the HMO revealed relatively stable and high satisfaction levels across all conflict zones during the war. In October 2023—which included three full weeks of active warfare—satisfaction in primary conflict zones was 84.1%, compared to 84.0% in the preceding month. Secondary conflict zones showed a minor increase from 86.6 to 86.7%, while low conflict zones experienced a slight decrease from 86.4 to 85.5%. These levels remained similarly stable in the months following the outbreak of war. Although the survey results refer to full calendar months and are not perfectly aligned with the study’s 30-day measurement windows starting on October 7, the consistent satisfaction levels before, during, and after the initial war period suggest that the rapid increase in telemedicine usage helped preserve not only access to care, but also patient-perceived quality of service under extreme conditions.

### Limitations

This study has several limitations. First, the data were collected from a single healthcare organization, which may limit generalizability. However, Maccabi Healthcare Services represents a significant portion of Israel’s healthcare system, providing a broad perspective. Second, the observational and retrospective design limits the ability to establish causality. To address this, we analyzed data across three distinct periods, including the month before the war, which coincided with holidays, and the same period from the previous year, to control for seasonal effects.

Third, while conflict zones were categorized based on geographical location and intensity, individual experiences within these zones may differ, potentially influencing healthcare-seeking behaviors. Finally, although the study focused on the first month of the war—an especially critical period marked by heightened trauma and fear due to the presence of terrorists inside Israel, the findings may not reflect long-term trends. To further expand on the value of these findings, future research should incorporate diagnostic-level data and patient outcomes to better distinguish between increased care demand and a shift in care modality. Such analyses will also help assess the effectiveness of telemedicine in maintaining quality of care in high-risk contexts. Despite these current limitations, the present study offers early empirical evidence to inform emergency preparedness, health system resilience, and targeted investments in digital health infrastructure.

## Conclusion

These findings highlight the adaptability of Israel’s HMO system in the face of significant challenges, while also identifying areas where improvements could be made to ensure that healthcare delivery remains resilient and equitable in future conflicts. The findings contribute to a growing understanding of how digital health can be utilized during crises and provide insights that could benefit healthcare systems globally.

The study suggests that investing in digital infrastructure and telemedicine capabilities is important for strengthening healthcare systems, particularly in regions vulnerable to conflict. By reinforcing these aspects, healthcare systems may be better equipped to maintain access to essential services, even in the most difficult circumstances.

## Data Availability

Access to the data may be granted upon reasonable request to the corresponding author, subject to approval by the Helsinki Committee of Maccabi Healthcare Services.
